# Evaluation of the SYNAPSE VINCENT for lateral lymph node dissection in rectal cancer with robotic surgery: a preliminary report

**DOI:** 10.1186/s12957-022-02532-2

**Published:** 2022-02-27

**Authors:** Nobuhisa Matsuhashi, Yuta Sato, Jesse Yu Tajima, Shigeru Kiyama, Takao Takahashi, Masashi Kuno, Masahide Endo, Masahiro Fukada, Chika Mizutani, Yoshihisa Tokumaru, Itaru Yasufuku, Tomonari Suetsugu, Yoshihiro Tanaka, Naoki Okumura, Katsutoshi Murase, Takuya Saiki, Kazuhiro Yoshida

**Affiliations:** 1 Department of Gastroenterological Surgery, Gifu University Graduate School of Medicine, Gifu, 501-1194 Japan; 2 Medical Education Development Center, Gifu University, Gifu, Japan

## Abstract

**Background:**

Even if 3D angiographic images of preoperative contrast-enhanced computed tomography (CT) are created, the coronal and axial sections can be unclear, and thus, it is difficult to achieve projection awareness similar to that of actual laparoscopic images. In recent years, the technology of analyzing and applying medical images has advanced, and surgical simulation and navigation have been widely used to improve the safety of surgical operations. It is important to understand pelvic anatomy in the area of rectal cancer, and use of the SYNAPSE VINCENT makes it possible to simulate the anatomy before surgery, which is very useful in educating surgeons and their assistants.

**Materials and methods:**

An important objective in surgery is to understand the anatomy of the external/internal iliac arteries and lymph nodes in lateral lymph node dissection (LLD) for rectal cancer. In this study, we explored the accuracy and usefulness of SYNAPSE VINCENT images of pelvic anatomy (especially vascular anatomy) analyzed preoperatively in two cases of LLD for rectal cancer in our department.

**Results:**

The patients were two men aged 73 and 57 years, respectively. Both patients underwent robotic abdominal perineal resection and LLD with neoadjuvant chemoradiotherapy. The operating times for LLD were 138 and 106 min, estimated blood loss was less than 10 mL and 20 mL, and the harvested lymph nodes were nos. 21 and 22, respectively. The SYNAPSE VINCENT could be used for simulation and navigation before and during surgery. For experienced surgeons, the system helped them carry out operations more accurately.

**Conclusion:**

In the future, surgical support using virtual reality, augmented reality, and mixed reality based on medical images will be useful and is expected to improve the safety, accuracy, and efficiency of surgery, which is extremely useful for both young and skilled surgeons preparing for difficult operations.

## Introduction

In recent years, advances have been made in the analysis and application of medical imaging, and the era of surgical simulation and navigation for improving the safety of surgical operations has arrived. In addition, mixed reality, augmented reality, and virtual reality technologies have become indispensable tools in medical image analysis to improve spatial awareness and to support imaging techniques. The SYNAPSE VINCENT (Fujifilm Medical Co., Ltd., Tokyo, Japan) is a three-dimensional (3D) image analysis system that allows 3D visualization of medical images for use in image diagnosis and surgical simulation.

In this context, the SYNAPSE VINCENT is actively used in the field of hepatobiliary and pancreatic surgery because it can create 3D computed tomography (CT) images for surgical support in a short time by applying image recognition technology used for face recognition in digital cameras. It is very important to understand the pelvic anatomy in rectal cancer surgery, and the SYNAPSE VINCENT makes it possible to simulate the anatomy before surgery, which is very useful not only for experienced surgeons but also surgeons in training.

## Material and methods

**Fig. 1:**
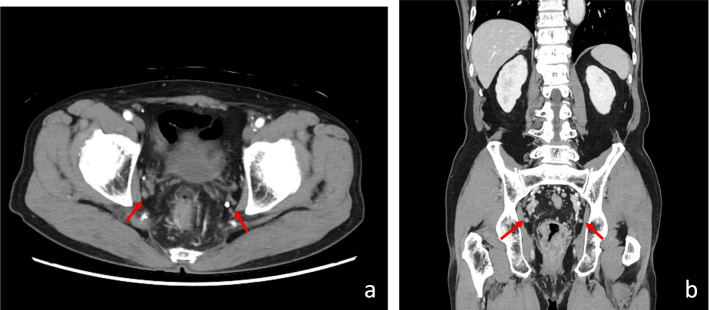
CT examination showed pelvic lymph node swelling in the axial plane (**a**) and the coronal plane (**b**) (red arrowhead)

**Fig. 2:**
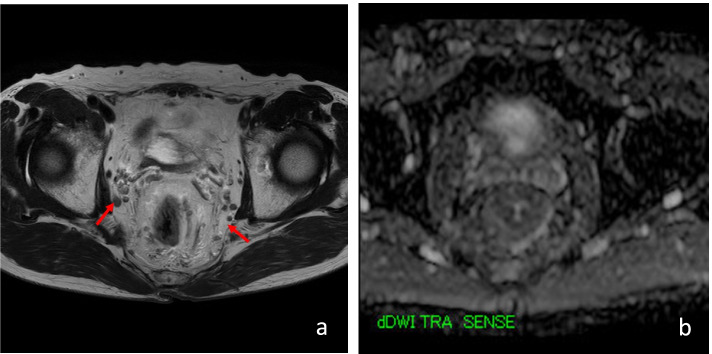
T2 and DWI MRI examinations showed pelvic lymph node swelling (red arrowhead)

Pelvic magnetic resonance imaging (MRI) and CT are performed in the preoperative evaluation of rectal cancer. From April 2012 to the present, preoperative dynamic contrast-enhanced CT (Fig. 1) and MRI (Fig. 2) were performed in our institution in all patients with rectal cancer, mainly lower rectal cancer, who were scheduled to undergo lateral lymph node dissection (LLD).

**Fig. 3:**
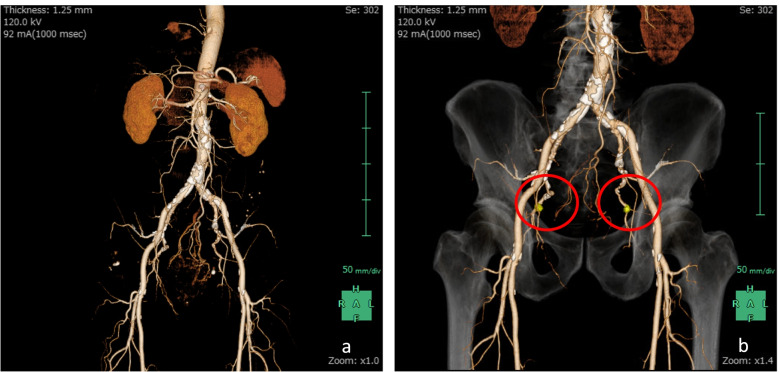
The locations of pelvic lymph node swelling were identified before surgery on 3D images using SYNAPSE VINCENT

In addition, we evaluated the anatomy of the pelvic cavity to clarify it. First, the paths of the internal iliac arteriovenous and external iliac arteriovenous systems were converted into coronal 3D images by the SYNAPSE VINCENT (Fig. 3). Second, swollen lymph nodes were also converted into 3D images to complete the preoperative schematic image. The surgeon trains for the surgery preoperatively by using the simulation images and then evaluates errors between the preoperative simulation and the actual surgery postoperatively. We performed a pilot trial aimed at evaluating the feasibility of using the SYNAPSE VINCENT in preoperative simulations and whether its use improved the safety of LLD with robotic surgery.

## Results

This pilot study evaluated the use of the SYNAPSE VINCENT in two male patients aged 73 and 57 years, respectively, between January 2021 and February 2021. Both patients underwent robotic abdominal perineal resection and LLD with neoadjuvant chemoradiotherapy (nCRT). The operating times for LLD were 138 and 106 min, estimated blood loss was less than 10 mL and 20 mL, and harvested lymph nodes were nos. 21 and 22, respectively.

**Fig. 4:**
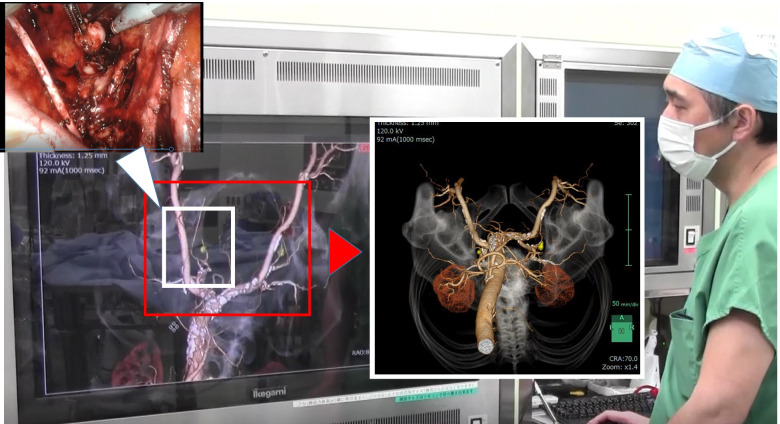
Pre- and intra-operative simulations using SYNAPSE VINCENT allow the surgeon to visualize the actual field of view

SYNAPSE VINCENT can be used for simulation and navigation before and during robotic surgery (Fig. 4). For experienced surgeons, it helps to perform lateral pelvic lymph node dissection. On the other hand, younger surgeons can learn anatomy and become familiar with this procedure and difficult cases more quickly even if they have not experienced many cases.

## Discussion

Navigation systems are used in the fields of orthopedic surgery and neurosurgery as surgical support systems because they can create 3D CT images in a short period of time by applying image recognition technology [1]. In gastrointestinal surgery and especially hepatobiliary and pancreatic surgery, the SYNAPSE VINCENT has been reported to be useful preoperatively in creating simulation and navigation images [2, 3]. In the area around the liver, the SYNAPSE VINCENT not only identifies the liver by using “liver anatomical analysis” on contrast-enhanced CT images, but it also applies this technology to the identification of blood vessels, thereby eliminating a complicated process and supporting faster image creation [4]. The system is capable of identifying the intricate vascular structures of the portal vein and hepatic artery and vein in a clear 3D format as well as recognizing the dominant region of each vessel and providing color-coded and segmented volumetric data [5]. This makes it possible to perform preoperative simulations with accurate, high-resolution images and is a useful clinical application in the operation of treatment plans such as for liver resection.

In the field of colorectal surgery, laparoscopic-assisted surgery for colorectal cancer has been increasing in recent years due to the widespread use of laparoscopic surgery [6]. Laparoscopy also allows precise manipulation of the surgical field due to the effects of magnification and proximity vision. However, the narrow field of view and the lack of tactile sensation are challenges to be overcome. To perform radical colorectal cancer surgery safely, it is extremely important to understand the preoperative locations and roots of the arteries. Recent advances in high-resolution, high-speed 3D-CT and image construction software have made it possible to construct arterial images minimally invasively within a short time and to understand the bifurcations of the arteries [7]. In colorectal cancer surgery, identification of the vessels feeding the tumor is very important in determining the area to be dissected. However, most previous studies have classified the bifurcation patterns of the superior and inferior mesenteric arteries from the main trunk, and no study has examined internal and external iliac arteries within the pelvic anatomy. The colorectal anatomy is not fixed due to its inherent organ specificity, and there can be a large difference in anatomical positioning depending on the angle of the obtained image [8]. Therefore, even if the central branches of the superior and inferior mesenteric arteries can be determined by preoperative image evaluation, identification of the peripheral vessels may not be as effective in preoperative simulations because of the movement of the intestines during the actual operation [9]. We considered preoperative simulations with SYNAPSE VINCENT to be most effective when they involve the pelvic vessels.

There are many anatomical variations in vascularization by the external and internal iliac arteries during LLD for rectal cancer that may cause unexpected anomalies during surgery. de Treigny et al. reported that it is important to understand that there is a difference in the male to female ratio of vascularization in the internal iliac artery region [10]. The anatomy for lymph node dissection of nodes #283 (obturator lymph node region) and #263d and #263p (internal iliac artery lymph node region distal and proximal) in the Japanese Classification of Colorectal, Appendiceal, and Anal Carcinoma is very difficult to understand, and the surgical technique is not easy.

In Japan, CRT is not used routinely; instead, LLD is performed to control disease on the pelvic sidewall. Sugihara et al. reported that the incidence of lateral lymph node metastasis in patients with T3 or T4 lower rectal cancer, located at or below the peritoneal reflection, was 18.1% [11]. Total mesorectal excision with lateral pelvic lymphadenectomy reduces the risk of pelvic recurrence, especially in radiologically positive cases. The main issue remains the risk of lateral pelvic lymph node metastases even after nCRT. The literature reports a high percentage (up to 30–40%) of pelvic lymph node involvement even after nCRT [12]. Anania et al. reported as an important objective the need for improved imaging techniques to accurately define a reliable cut-off size and describe radiological abnormalities that accurately predict involvement of pelvic lymph nodes [13]. In addition, even if 3D angiographic images are created with preoperative contrast-enhanced CT, the coronal and axial sections alone may not be clear because there is a significant difference in the surgeon’s field of view during the actual surgery. Sun et al reported that this meta-analysis suggests that robotic-low anterior resection (LAR) is associated with a shorter hospital stay, lower conversion rate, lower rate of circumferential margin involvement, and lower overall complication rate than laparoscopic-LAR. There were no differences in operative time and the number of lymph nodes removed [14]. Clinical studies using surgical techniques are difficult to derive statistical significance in a small number of cases.

This pilot trial was aimed at evaluating the feasibility of the SYNAPSE VINCENT to provide preoperative simulations that are useful and improve the safety of LLD with robotic surgery. As a result, robotic rectal cancer surgery with preoperative CRT (a high-complexity surgery) was safe and feasible in terms of operative time, blood loss, and number of lymph nodes dissected. Preoperative simulations using the SYNAPSE VINCENT will allow the surgeon to visualize the field of view as it would be during the actual surgery and will enable the surgeon to perform pelvic dissection without leaving behind any lymph nodes, especially in the deep pelvic cavity. In the future, we expect to expand our analysis with definitive results regarding safety, costs, feasibility, and oncological outcome, concerning the evaluation of the use of SYNAPSE VINCENT in LLD with robotic surgery.

## Conclusion

It is expected that digital transformation will become more widespread and evolve in the field of surgery. It will be very useful for skilled surgeons to prepare for difficult operations, and young surgeons can use digital transformation for simulation training without the need for cadaver training. It is also expected to result in an excellent learning curve for surgeons.

## Data Availability

Not applicable.
